# Different Starch Sources Affect the Growth Performance and Hepatic Health Status of Largemouth Bass (*Micropterus salmoides*) in a High-Temperature Environment

**DOI:** 10.3390/ani13243808

**Published:** 2023-12-10

**Authors:** Dongyu Huang, Jiaze Gu, Chunyu Xue, Lu Zhang, Xiaoru Chen, Yongli Wang, Hualiang Liang, Mingchun Ren

**Affiliations:** 1 Key Laboratory of Integrated Rice-Fish Farming Ecology, Ministry of Agriculture and Rural Affairs, Freshwater Fisheries Research Center, Chinese Academy of Fishery Sciences, Wuxi 214081, China; 2 Wuxi Fisheries College, Nanjing Agricultural University, Wuxi 214081, China; 3 Tongwei Agricultural Development Co., Ltd., Key Laboratory of Nutrition and Healthy Culture of Aquatic Livestock and Poultry, Ministry of Agriculture and Rural Affairs, Healthy Aquaculture Key Laboratory of Sichuan Province, Chengdu 610093, China

**Keywords:** wheat starch, growth performance, hepatic health status, high temperature, largemouth bass

## Abstract

**Simple Summary:**

This experiment was conducted to investigate the effects of different starch sources on the growth performance and hepatic health of largemouth bass in a high-temperature environment. The largemouth bass were fed the experimental diet for 45 days. In this study, wheat starch resulted in better growth performance in largemouth bass in a high-temperature environment and had a positive effect on the antioxidant status of largemouth bass liver under high-temperature conditions. In conclusion, wheat starch is more suitable than corn starch, tapioca starch, sweet potato starch, and potato starch for largemouth bass, based on the growth performance, survival rate, liver pathology, hepatic antioxidant capacity, and immune response analyses.

**Abstract:**

The experiment was designed to investigate the effects of different starch types on the growth performance and liver health status of largemouth bass in a high-temperature environment (33–35 °C). In this study, we designed five diets using corn starch (CS), tapioca starch (TS), sweet potato starch (SPS), potato starch (PS), and wheat starch (WS) as the starch sources (10%). We selected 225 healthy and uniformly sized largemouth bass (199.6 ± 0.43 g) and conducted the feeding experiment for 45 days. The results showed that the WS group had the highest WGR, SGR, and SR and the lowest FCR. Among the five groups, the WS group had the highest CAT activity, SOD activity, and GSH content, while the SPS group had the highest MDA content. Furthermore, oil red O staining of liver samples showed that the TS group had the largest positive region, indicating high lipid accumulation. Lastly, the gene expression results revealed that compared with the WS group, the CS, TS, and SPS groups showed suppressed expression of *nrf2*, *keap1*, *cat*, *sod*, *gpx*, *il-8*, and *il-10*. Therefore, our results demonstrated the effect of different starch sources on largemouth bass growth performance and hepatic health in a high-temperature environment.

## 1. Introduction

Global warming has intensified in recent years, and climate models predict that temperatures will continue to rise approximately 5 °C by 2100 [1]. China is also sensitive to global warming, with an average temperature rise of 0.22 °C per decade [2]. The warmer temperatures over time will inevitably lead to higher water temperatures, and increased water temperature adversely affects the growth and survival of several fish species [3,4]. Therefore, in recent years, researchers have paid increasing attention to the heat stress response mechanisms in fish [5]. Water conditions, especially water temperature, affect the growth, reproduction, immune regulation, and physiological activities of fish [6,7,8]. Moreover, persistent high temperatures may reduce the growth and reproductive ability of fish as well as deteriorate their health, eventually leading to their death [9,10].

Carbohydrates are the most economical energy source for animals, and the addition of appropriate amounts of carbohydrates to fish feed can serve to conserve protein [11,12], which not only reduces the cost of feed but also activates amino acids to promote the growth of fish. However, studies have shown that carbohydrate is not an essential nutrient for fish and that fish consuming low-carbohydrate diets continue to grow and survive [13,14], possibly due to their ability to form glucose via gluconeogenesis [15]. Nevertheless, a study found that compared to a low-carbohydrate diet, an appropriate carbohydrate-supplemented diet significantly increased the growth rates of some carnivorous fish [16]. The carbohydrate-utilization ability of fish varies from species to species and is influenced by the level and structure of carbohydrates in the feed [17]. Based on their structure, carbohydrates can be categorized into monosaccharides, disaccharides, oligosaccharides, and polysaccharides. Currently, the most widely used carbohydrate source in fish feed is starch [18], and the ratio of straight-chain to branched-chain starch significantly affects its digestion and absorption in fish [19]. Rainbow trout (*Oncorhynchus mykiss*) can utilize branched-chain starch more effectively than straight-chain starch [20], while tongue-tooth bass (*Dicentrarchus labrax*) showed no significant difference in the utilization of straight-chain or branched-chain starch [21]. Therefore, the study of the aptitude of fish for starch sources is of great importance in nutrition and feed science.

Largemouth bass (*Micropterus salmoides*) is an economic carnivorous fish with poor carbohydrate-utilization ability [22]. Studies have indicated that largemouth bass can grow in 10–32 °C water temperatures, but the optimum growth temperature of largemouth bass is from 26 to 29 °C [23,24]. The increase in water temperature due to global warming could lead to high mortality of largemouth bass cultured during the summer [25,26]. Temperature directly affects the body metabolism of aquatic animals, including rainbow trout [7], grass carp (*Ctenopharyngodon idella*) [8], and largemouth bass [27]. These studies indicated that the metabolism and immune regulation could be disturbed in high-temperature environments [27], which may be one of the reasons for fish death. Many studies have shown that high-carbohydrate diets can affect largemouth bass health. Zhao et al. [28] found that compared to a low-carbohydrate diet (9.66%), a high-carbohydrate diet (19.11%) led to the accumulation of glycogen and lipid droplets and the occurrence of vacuolation in liver and induced antioxidant response through the glutathione antioxidant system. In addition, Zhao et al. [29] also revealed that a high-carbohydrate diet (17%) impairs the intestinal barrier in juvenile largemouth bass, which was not found in a low-carbohydrate diet (7%). However, whether the selection of different starch sources in largemouth bass diets under high-temperature environments affects growth performance and liver health has not been reported in studies. Therefore, five experimental diets were prepared with five kinds of starch (corn starch, tapioca starch, sweet potato starch, potato starch, and wheat starch), and the effects of different starch sources in the diets on the growth and liver histology under high temperature were evaluated to find the optimal starch sources that are beneficial to the liver health of largemouth bass under high-temperature conditions.

## 2. Materials and Methods

### 2.1. Experimental Diets

In this study, we formulated five isonitrogenous and isolipid diets (Table 1) using fish meal, blood meal, enzymatic hydrolysis of poultry by-products, and soybean meal as the main protein sources; fish oil and shrimp paste as the main lipid sources; and corn starch (CS), tapioca starch (TS), sweet potato starch (SPS), potato starch (PS), and wheat starch (WS) as the different starch sources. According to a previous study [28], a low-carbohydrate diet (9.66%) was better for the health of largemouth bass, and thus starch levels were set at 10% for this experiment. The ingredients were crushed through an 80-mesh sieve, weighed, and mixed using a step-by-step expansion method. Then, fish oil and water (25%, mass fraction) were added and mixed using a blender to obtain the expanded pellet feed (6 mm) using a dry-expanding machine. The pellets were air-dried in an air-conditioned room, packed in sealed bags, and stored in a refrigerator at −20 °C.

### 2.2. Experimental Design and Management

Largemouth bass of the same genetic background were selected. They were all obtained from Nanquan Base of the Freshwater Fisheries Research Center (Wuxi, China) and temporarily reared for 2 weeks in outdoor cages in a pond (0.2 hectares). At the beginning of the experiment, the fish were fasted for 24 h, and 225 healthy and uniformly sized fish (199.6 ± 0.43 g) were randomly divided into 15 floating cages (1 × 1 × 1 m) (n = 15/cage) in the pond (0.2 hectares), and 15 cages were divided into five groups (CS group, TS group, SPS group, PS group, and WS group) with three replicates per group. The fish were fed daily at 6:00 and 18:00 until apparent satiation. The aquaculture experiments were conducted for 45 days at Nanquan Base (31.568° N, 120.299° E; Freshwater Fisheries Research Center, Chinese Academy of Fisheries Sciences). The water temperature varied compared with the usual temperature and ranged from 33–35 °C during the feeding trial, and the pond was equipped with oxygenation equipment to ensure adequate oxygenation. The dissolved oxygen content was 6.52 ± 0.98 mg/L during the experimental period.

### 2.3. Sample Collection

The fish were fasted for 24 h after the feeding experiment and counted to record the survival rate (SR). Thereafter, the fish were weighed to calculate the weight gain rate (WGR) and specific growth rate (SGR). The feed intake was recorded during feeding to calculate the feed conversion ratio (FCR) [30].

Two fish from each cage were randomly selected for whole-body composition analysis. Additionally, three fish were randomly selected from each cage for the analysis of plasma biochemical indices. Blood was drawn from the tail vein with a 2.5 mL sterile syringe, transferred to 1.5 mL centrifuge tubes, and centrifuged at 3500 rpm for 10 min (Eppendorf 5424R, Eppendorf AG, Hamburg, Germany) at 4 ℃ to obtain the plasma. The liver tissues were stored in cryopreservation tubes and stored at −80 ℃ for subsequent enzyme activity and genetic analyses. In addition, according to a previous method of tissue preservation, a portion of liver tissue was preserved in 4% paraformaldehyde for histological analysis [21].

### 2.4. Analysis of Nutrient Composition, Plasma Biochemical Indices, and Hepatic Antioxidant Indices

The nutrient composition of the experimental diets and the whole fish were analyzed using the Association of Official Analytical Chemists (AOAC) [31] method. Analysis of the plasma biochemical indices was conducted using an automatic biochemical analyzer (Mindray BS-400) and the corresponding detection reagents purchased from Mindray Medical International Ltd. (Shenzhen, China). Hepatic antioxidant enzyme activity wase determined by enzyme labeling using the corresponding kits (Jian Cheng Bioengineering Institute (Nanjing, China)), and utilizing a spectrophotometer (Thermo Fisher Multiskan GO, Shanghai, China). The kits, equipment, and methods used for the above analyses are listed in Table 2.

### 2.5. Histological Analysis

The liver samples were subjected to oil red O staining as described previously [32]. Briefly, the fresh tissues were fixed in 4% paraformaldehyde, trimmed, and dehydrated. Thereafter, the samples were embedded in an optimal cutting temperature compound, frozen, and sectioned. Lastly, the sections were stained with oil red O working solution and observed under a microscope (Axioplan 2, Oberkochen, Germany).

### 2.6. RNA Extraction and qRT-PCR Assay

Total RNA was extracted from the liver tissues using TRIzol reagent, and its integrity was examined by electrophoresis on a 1.0% denaturing agarose gel. The purity and concentration of RNA were determined using NanoDrop 2000 (Thermo Scientific, New York, NY, USA). Eligible RNA samples were treated with RNA-free DNase reagent to remove DNA contaminants.

Gene expression was determined by qRT-PCR assay using specific primers (Table 3). The antioxidant (*nrf2*, *keap1*, *cat*, *sod*, *gpx*) and immune (*nf-κb*, *tnf-α*, *il-8*, *il-10*)-related genes were selected to detect their relative expression levels. *β-actin* was used as the internal reference gene. The PCR reaction was performed using the HiScript^®^II One Step qRT–PCR SYBR Green Kit (Vazyme Biotech Co., Ltd., Nanjing, China) on a Bio-Rad CFX96 real-time fluorescence quantifier (Shanghai, China). The relative standard curve method was used to calculate the relative mRNA expression of the target genes.

### 2.7. Statistical Analysis

SPSS 25.0 and GraphPad Prism 7.0 software were used for statistical analysis and graphing. The data were subjected to normality and homogeneity tests where necessary. All data were square root-transformed when necessary to better meet assumptions of equal variance and normality. The results are expressed as means ± standard error of mean (SEM), and the experimental data (means ± SEM) were analyzed using SPSS 25.0 statistical software for one-way analysis of variance (ANOVA). When the difference was significant (*p* < 0.05), Duncan’s multiple comparisons were performed.

## 3. Results

### 3.1. Growth Performance

The effects of dietary starch sources on the growth performance and morphological indices of largemouth bass are shown in Table 4. The FBW, WGR, and SGR of the PS and WS groups were significantly higher than those of the TS group (*p* < 0.05), with no significant differences compared with the CS and SPS groups (*p* > 0.05). In addition, the FCR of the PS and WS groups was lower than those of the TS group (*p* < 0.05).

### 3.2. Whole-Body Composition

The effects of dietary starch sources on the whole-body composition of largemouth bass are shown in Table 5. There were no significant changes in body moisture, crude lipid, crude protein, and crude ash contents among any starch source treatment groups (*p* > 0.05).

### 3.3. Plasma Biochemical Indices

The effects of dietary starch sources on the plasma biochemical indices of largemouth bass are shown in Table 6. It was found that the TP contents of the SPS, PS, and WS groups were significantly higher than that of the TS group (*p* < 0.05), and the ALT activities of the PS and WS groups were significantly lower than that of the CS and TS groups (*p* < 0.05). In addition, plasma ALB contents and AST activities were not affected by the starch sources (*p* > 0.05).

### 3.4. Hepatic Antioxidant Indices

Table 7 presents the antioxidant indices of largemouth bass. The highest CAT activity was observed in the WS group, which was greater than that of the CS group (*p* < 0.05). The highest SOD activity was also observed in the WS group, which was significantly higher than that of the CS, TS, and SPS groups (*p* < 0.05). Moreover, the MDA content of the SPS group was remarkably higher than that of the WS group (*p* < 0.05). In addition, the GSH content was observed in the WS group, which was significantly higher than that of the SPS group (*p* < 0.05).

### 3.5. Liver Pathology Analysis

**Figure 1:**
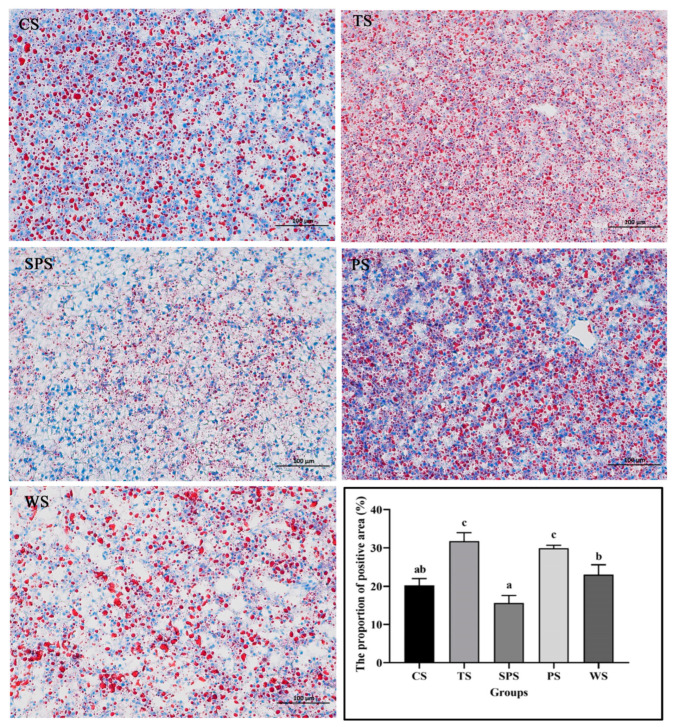
Oil red O-stained sections and the proportion of the positive area of largemouth bass liver (200×). Values with different superscripts are significantly different (*p* < 0.05).

The effects of dietary starch sources on liver histomorphology are shown in Figure 1. By oil red O staining, the lipid droplets are orange or bright red, the nucleus is blue, and the rest of the cell is nearly colorless. In addition, the results of the proportion of positive areas in the different starch groups showed that the positive areas of the TS and PS groups were larger than those of other groups (*p* < 0.05).

### 3.6. qRT-PCR Assay

**Figure 2:**
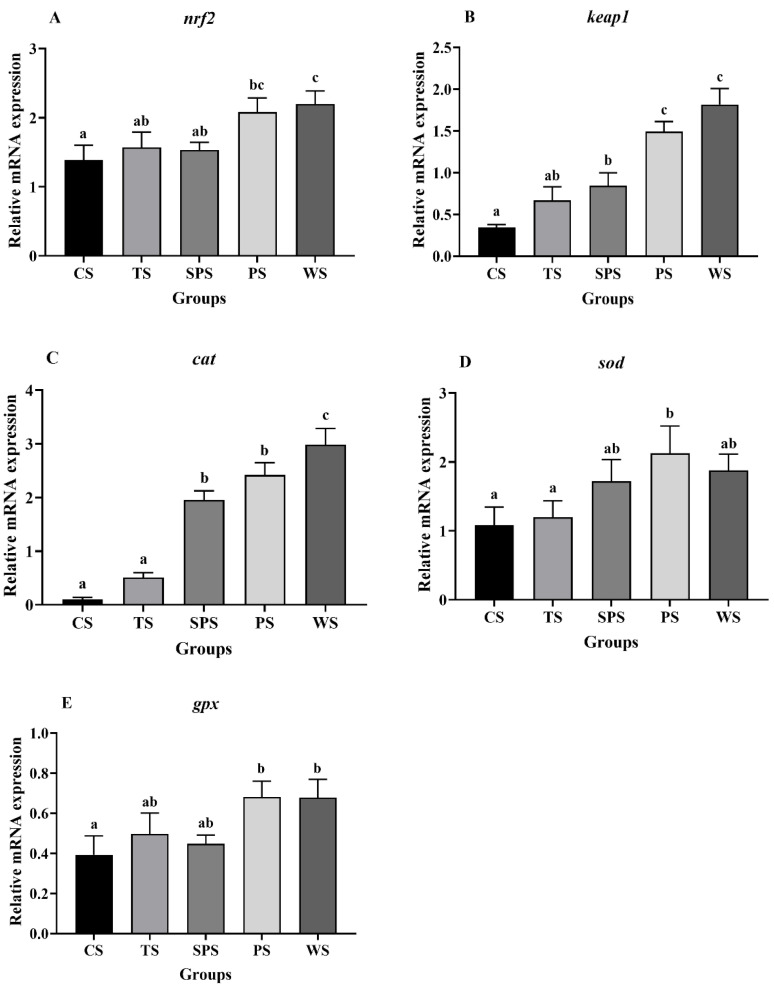
Relative expressions of Nrf2-Keap1 signaling pathway ((**A**) *nrf2*, (**B**) *keap1*, (**C**) *cat*, (**D**) *sod*, (**E**) *gpx*). Values with different superscripts are significantly different (*p* < 0.05).

The relative gene expression of the Nrf2-Keap1 signaling pathway is shown in Figure 2. In comparison with the CS, TS, and SPS groups, the WS group significantly increased the *nrf2* mRNA expression level (*p* < 0.05, Figure 2A), and the PS and WS groups increased the *keap1* mRNA expression levels compared to other starch groups (*p* < 0.05, Figure 2B). In addition, the *cat* mRNA levels were significantly upregulated in the WS group, and were higher than those of other groups (*p* < 0.05, Figure 2C). The *sod* mRNA levels were significantly upregulated in the PS group, and were larger than those of the CS and TS groups (*p* < 0.05, Figure 2D). The *gpx* mRNA levels were significantly upregulated in the PS and WS groups, and were larger than that of the CS group (*p* < 0.05, Figure 2E).

**Figure 3:**
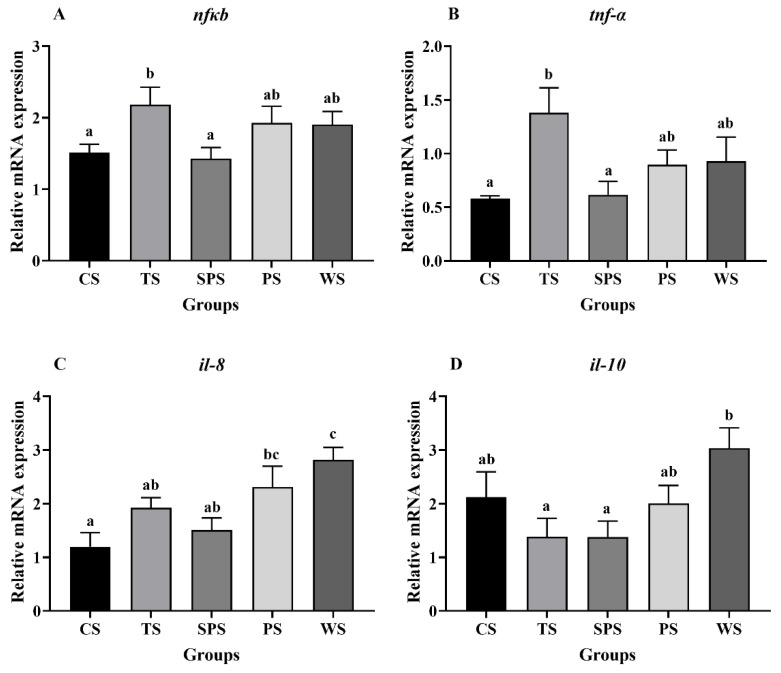
Relative expressions of NF-κB signaling pathway ((**A**) *nfκB*, (**B**) *tnf-α*, (**C**) *il-8*, (**D**) *il-10*). Values with different superscripts are significantly different (*p* < 0.05).

The relative gene expression of the NF-κB signaling pathway is shown in Figure 3. The *nfκb* and *tnf-α* mRNA expression levels of the TS group were significantly upregulated compared to those of the CS and TS groups (*p* < 0.05, Figure 3A,B). In addition, in comparison with the CS, TS, and SPS groups, the WS group showed significantly increased *il-8* mRNA expression levels (*p* < 0.05, Figure 3C), and in the WS group significantly increased *il-10* mRNA expression levels compared to the TS and SPS groups (*p* < 0.05, Figure 3D).

## 4. Discussion

In recent years, high-temperature stress on fish has been increasing day by day as global warming results in water temperature increases [2]. Water temperature is one of the most critical environmental factors in aquaculture: when the temperature exceeds the optimal growth temperature, the growth rate decreases with the increase in temperature [35]. Furthermore, high-temperature stress can reduce the immune ability of the fish body, and will produce diseases and even death [36]. To date, studies on the effects of different starch sources on the growth and liver health of largemouth bass at high temperatures have not been reported, which is an area worth investigating.

Previous studies found that starch from different sources affects the growth of several aquaculture species, including swimming crab (*Portunus trituberculatus*) [37], blunt snout bream (*Megalobrama amblycephala*) [23], rohu (*Labeo rohita*) [38], and juvenile yellow perch (*Perca flavescens*) [39]. In addition, studies found that carbohydrate utilization increases in fish in high-temperature environments and the fish will show different growth performances for different starch sources [38]. The same species can also differ in the digestibility of different starch sources due to differences in the content of indigestible polysaccharides in the sources, causing differences in growth performance [40,41]. Our present results showed that different starch sources in the feed significantly affected the growth performance and feed utilization of largemouth bass in a high-temperature environment. Compared with the CS, the proportion of amylose in other starches is higher, so it may cause better growth performance in largemouth bass under high-temperature environments, especially WS and PS. Li et al. [22] reported that a high amylopectin ratio in starch can promote the growth of carnivorous fish, which may be caused by excessive amylopectin hydrolysis after ingestion, which is not conducive to the normal growth of largemouth bass. Although high temperatures can reduce fish digestibility [42], at the same high temperatures, starch with a high direct branch ratio leads to slower digestion, which may be more conducive to improving growth performance [43,44].

Our results revealed that the whole-body composition was not significantly different among the five experimental groups, which is consistent with previous findings on largemouth bass and other carnivorous fish. Song et al. [45] found that largemouth bass fed different starch sources, including WS, pea starch, cassava starch, and high-amylose CS, showed no significant differences in whole-body composition. Additionally, Dang et al. [46] reported that cassava starch, WS, pea starch, and CS did not significantly affect the whole-body composition of rainbow trout.

The liver is an important immune organ, and excessive carbohydrates in fish feed can adversely affect liver health by inducing metabolic disorders and liver tissue damage in some fish species [47,48]. Additionally, the type (or source) of carbohydrates in fish feed also affects their utilization and tolerance by carnivorous fish [16]. In this study, we found that different starch sources affected the liver tissue of largemouth bass to varying degrees. Although oil red O staining showed liver steatosis in all the groups, the positive regions in the hepatic samples of TS and PS groups were significantly larger than those of the other groups, thus reflecting the poor physiological state of the liver in these groups. This may be due to the conversion of the TS and PS carbohydrates into lipids and their subsequent accumulation in the liver. Du [49] found that the accumulation of lipids in the liver is an indirect reaction to liver injury and that it can reduce growth performance, immunity, and stress tolerance. Plasma ALT and AST are important indicators of liver function, and their overexpression indicates liver damage [50]. High temperatures induce increased plasma AST and ALT activities [51]. Our results revealed significantly increased plasma ALT activities in the TS and CS groups, indicating severe liver damage in these groups. These results indicated that TS and CS diets impaired the normal metabolic functioning of the largemouth bass liver, which may be further aggravated by the high-temperature environment.

Nrf2 is recognized as a dominant regulator of oxidative stress in cells and Nrf2-Keap1 controls the gene expression of several detoxification enzymes and antioxidant proteins [52]. In this experiment, *nrf2* mRNA expression was upregulated in the PS and WS groups, concomitant with the upregulation of *keap1* mRNA. Normally, Keap1 is an important factor interacting with the Neh2 de electron domain of Nrf2 and plays an important negative regulatory role on Nrf2 [53]. Wang et al. [54] found that the changes in Keap1-Nrf2 signaling pathway expression in dairy cows induced by heat stress and the *keap1* and *nrf2* mRNA levels in the liver were increased remarkably during heat stress, indicating that oxidative stress occurred. Thus, our experimental results show that the mechanism of antioxidant damage is active in the PS and WS groups under a high-temperature environment. Starch with a high direct branch ratio improved antioxidant capacity in animals [55], which could explain the reason for our experimental results. Liu et al. [56] also found that antioxidant genes (*nrf2*, *cat*) were upregulated in largemouth bass and that high amylopectin improved intestinal antioxidant capacity. In mice, high amylopectin also significantly upregulated the expression of the *nrf2* gene [57]. These results suggested that high amylopectin can enhance antioxidant enzyme activities by regulating the expression level of the *nrf2* gene. In addition, the transcriptional expression levels of downstream antioxidant elements including *cat*, *sod*, and *gpx* were activated in the PS or WS group. Moreover, heat stress can increase the reactive oxygen species in vivo, but with the continuous accumulation of oxygen free radicals in vivo, the activity of antioxidant enzymes is not enough to inhibit oxidative damage in cells, thus reducing the activity of antioxidant enzymes in vivo [58]. In this study, some antioxidant enzymes (CAT, SOD, and GSH) showed their highest activities in the WS group, which was consistent with the expression of antioxidant genes. Furthermore, MDA contents reflect the severity of free radical attacks on body cells [59], and our experiment results showed that CS and WS did not cause a significant accumulation of MDA content compared to other starch groups. Altogether, these findings indicate that compared with the other starch sources, WS has a positive effect on the antioxidant status of largemouth bass liver in high-temperature environments.

The Nrf2 signaling pathway can synergistically interact with the NF-κB signaling pathway to enhance overall immune tolerance by inducing the expression of anti-inflammatory genes [60]. Straight-chain starch has fewer branches that can form resistant starch with complex crystal structures, and the appropriate resistant starch can reduce the inflammatory response and improve the body’s resistance to disease [61]. In this study, we found that the expression of *nf-κb* and the downstream proinflammatory factor *tnf-α* was upregulated in the TS group, indicating that liver injury stimulates the expression of TNF-α to clear damaged hepatocytes and promote healing [62]. The gene expression results were consistent with the mortality results, suggesting that the TS group had low immunity and the fish susceptible to heat stress damage. Furthermore, the expression of the downstream anti-inflammatory factors *il-8* and *il-10* was the highest in the WS group, indicating that compared to the other starch sources, WS promotes immune response in largemouth bass in high-temperature environments. In a human intestinal fermentation model, it has been suggested that increased butyrate content due to a high-straight-chain starch diet is also an important factor in the immune enhancement of the animals, as butyrate can inhibit the NF-κB signaling pathway by weakening histone deacetylase, thereby downregulating the production of proinflammatory cytokines [63]. In the present study, similarly, the WS with a high direct branch ratio was found to improve hepatic immune function. Studies in turbot (*Paralichthys olivaceus*) also found that high straight-chain starch significantly upregulated the expression of immune-related genes [64]. Furthermore, increased plasma ALB and TP levels indicate enhanced innate immunity in the body [65,66]. Our results revealed that TP was significantly lower in the TS group compared to the other starch groups, indicating that the use of TS in fish feed is not conducive to the enhancement of immunity in largemouth bass under high-temperature conditions and that the use of WS is more conducive to the activation of the immune response in largemouth bass.

## 5. Conclusions

In conclusion, our study found that under high-temperature conditions, based on growth performance, starch sources were prioritized in the order of wheat starch, potato starch, corn starch, sweet potato starch, and tapioca starch. Based on liver pathology, starch sources were prioritized in the order of sweet potato starch, corn starch, or wheat starch, followed by potato starch and tapioca starch; based on hepatic antioxidant capacity, and immune response analyses, starch sources were prioritized in the order of wheat starch or potato starch, followed by corn starch, sweet potato starch, and tapioca starch. This study demonstrated the effect of different starch sources on largemouth bass growth performance and hepatic health in a high-temperature environment, provides guidance for the selective use of starch sources in aquafeed, and offers some help for subsequent production practices.

## Figures and Tables

**Table 1:** Composition of the experimental diets (% dry matter).

Diet	CS	TS	SPS	PS	WS
Fish meal ^1^	46.00	46.00	46.00	46.00	46.00
Blood meal ^1^	2.00	2.00	2.00	2.00	2.00
Soybean meal ^1^	14.00	14.00	14.00	14.00	14.00
Corn gluten meal ^1^	4.00	4.00	4.00	4.00	4.00
Enzymatic hydrolysis of poultry by-products ^1^	6.00	6.00	6.00	6.00	6.00
Rice bran	8.35	8.35	8.35	8.35	8.35
Shrimp paste	2.00	2.00	2.00	2.00	2.00
Fish oil	3.90	3.90	3.90	3.90	3.90
Vitamin premix ^3^	1.00	1.00	1.00	1.00	1.00
Mineral premix ^3^	1.00	1.00	1.00	1.00	1.00
Calcium dihydrogen phosphate	1.20	1.20	1.20	1.20	1.20
Vitamin C	0.05	0.05	0.05	0.05	0.05
Choline chloride	0.50	0.50	0.50	0.50	0.50
Corn starch ^2^	10.00				
Tapioca starch ^2^		10.00			
Sweet potato starch ^2^			10.00		
Potato starch ^2^				10.00	
Wheat starch ^2^					10.00
*Analyzed proximate composition*					
Crude protein (%)	48.50	49.06	48.73	48.96	48.99
Crude lipid (%)	10.44	10.01	10.41	10.57	10.52

^1^ Fish meal obtained from Wuxi Tongwei Feedstuffs Co., Ltd., Wuxi, China, crude protein 66.7%, crude lipid 9.5%; Blood meal obtained from Wuxi Tongwei Feedstuffs Co., Ltd., Wuxi, China, crude protein 90.7%; soybean meal obtained from Wuxi Tongwei Feedstuffs Co., Ltd., Wuxi, China, crude protein 50.8%, crude lipid 4.3%; corn gluten meal obtained from Wuxi Tongwei Feedstuffs Co., Ltd., Wuxi, China, crude protein 59.2%, crude lipid 3.3%; enzymatic hydrolysis of poultry by-products obtained from Wuxi Tongwei Feedstuffs Co., Ltd., Wuxi, China, crude protein 84.6%, crude lipid 1.0%. ^2^ Corn starch, tapioca starch, sweet potato starch, potato starch, and wheat starch obtained from Wuxi Tongwei Feedstuffs Co., Ltd. ^3^ Mineral premix and vitamin premix obtained from Wuxi Hanove Animal Health Products Co., Ltd., Wuxi, China.

**Table 2:** The chemical analyses used in the experiments.

Items	Methods	Assay Kits/Testing Equipment
Plasma biochemistry parameters		
TP	International Federation of Clinical Chemistry recommended	Assay kits purchased from Mindray Medical International Ltd. (Shenzhen, China); Mindray BS-400 automatic biochemical analyzer (Mindray Medical International Ltd., Shenzhen, China).
ALB		
ALT		
AST		
Enzyme activity parameters		
MDA	TBA method	Assay kits purchased from Jian Cheng Bioengineering Institute (Nanjing, China); Spectrophotometer (Thermo Fisher Multiskan GO, Shanghai, China).
CAT	Ammonium molybdenum acid method	
SOD	WST-1 method	
GSH	Microplate method	

TP: total protein, ALB: albumin, ALT: alanine transaminase, AST: aspartate aminotransferase, CAT: catalase, SOD: superoxide dismutase, MDA: malondialdehyde, GSH: glutathione.

**Table 3:** The specific primers for the reference gene and target genes.

Genes	Forward Primer (5′–3′)	Reverse Primer (5′–3′)	Reference
*nrf2*	AGAGACATTCGCCGTAGA	TCGCAGTAGAGCAATCCT	NM_212855.2
*keap1*	CGTACGTCCAGGCCTTACTC	TGACGGAAATAACCCCCTGC	XP_018520553.1
*cat*	CTATGGCTCTCACACCTTC	TCCTCTACTGGCAGATTCT	MK614708.1
*sod*	TGGCAAGAACAAGAACCACA	CCTCTGATTTCTCCTGTCACC	Gu et al., 2022 [33]
*gpx*	GAAGGTGGATGTGAATGGA	CCAACCAGGAACTTCTCAA	MK614713.1
*nfκB*	CCACTCAGGTGTTGGAGCTT	TCCAGAGCACGACACACTTC	XP_027136364.1
*tnf-α*	CTTCGTCTACAGCCAGGCATCG	TTTGGCACACCGACCTCACC	Gu et al., 2022 [33]
*il-8*	TCGGTCCTCCTGGGTGAAAA	GTGCTCCTTCCTGCTGATGTA	ASK51661.1
*il-10*	CGGCACAGAAATCCCAGAGC	CAGCAGGCTCACAAAATAAACATCT	Yang et al., 2020 [34]
*β-actin*	CCACCTTCAACAGCATCA	AGCCTCCAATCCATACAGA	MH018565.1

*nrf2*, Nuclear factor erythroid 2-related factor 2; *keap1*, Kelch-like ECH-associated protein1; *cat*, catalase; *sod*, superoxide dismutase death domain; *gpx*, glutathione peroxidase; *nf-κb*, nuclear factor kappa B; *tnf-α*, tumor necrosis factor-α; *il-8*, interleukin 8; *il-10*, interleukin 10.

**Table 4:** Effect of different starch sources on growth performance of largemouth bass under high-temperature environment.

Groups	IBW (g) ^1^	FBW (g) ^2^	WGR (%) ^3^	SGR (%/Day) ^4^	FCR ^5^	SR (%) ^6^
Corn starch	198.89 ± 0.59	254.85 ± 6.08 ^ab^	28.12 ± 2.77 ^ab^	0.55 ± 0.05 ^ab^	1.53 ± 0.05 ^bc^	71.1 ± 2.22
Tapioca starch	198.89 ± 0.89	243.64 ± 7.44 ^a^	22.53 ± 4.21 ^a^	0.45 ± 0.08 ^a^	1.69 ± 0.13 ^c^	84.4 ± 8.01
Sweet potato starch	200.44 ± 0.80	250.75 ± 1.50 ^ab^	25.10 ± 1.09 ^ab^	0.50 ± 0.02 ^ab^	1.39 ± 0.12 ^abc^	68.9 ± 2.22
Potato starch	199.56 ± 0.97	271.19 ± 1.91 ^b^	35.89 ± 0.34 ^b^	0.68 ± 0.01 ^b^	1.20 ± 0.02 ^ab^	73.3 ± 3.85
Wheat starch	200.22 ± 1.60	272.85 ± 7.85 ^b^	36.26 ± 3.55 ^b^	0.69 ± 0.06 ^b^	1.13 ± 0.04 ^a^	84.4 ± 8.01
*p*-value	0.738	0.016	0.018	0.022	0.005	0.202

Data are expressed as means with SEM. Values with different superscripts are significantly different (*p* < 0.05). ^1^ IBW: initial body weight; ^2^ FBW: final body weight; ^3^ weight gain rate (WGR) (%) = 100 × (final body weight (g) − initial body weight (g))/initial body weight (g); ^4^ specific growth rate (SGR) (% day^−1^) = 100 × ((In (final body weight (g)) − In (initial body weight (g))/days]; ^5^ feed conversion ratio (FCR) = dry feed fed (g)/(final body weight (g) − initial body weight (g)); ^6^ survival rate (SR) (%) = 100 × (survival fish number/total fish number).

**Table 5:** Effect of different starch sources on whole-body composition of largemouth bass under high-temperature environment.

Groups	Moisture (%)	Crude Protein (%)	Crude Lipid (%)	Crude Ash (%)
Corn starch	67.97 ± 0.16	16.88 ± 0.17	8.56 ± 0.29	4.94 ± 0.07
Tapioca starch	67.35 ± 0.94	16.52 ± 0.46	8.71 ± 0.08	4.76 ± 0.25
Sweet potato starch	66.65 ± 0.67	16.72 ± 0.37	8.87 ± 0.47	5.06 ± 0.25
Potato starch	67.54 ± 0.58	17.35 ± 0.38	8.10 ± 0.30	5.07 ± 0.09
Wheat starch	67.29 ± 0.18	17.29 ± 0.17	8.33 ± 0.29	4.83 ± 0.09
*p*-value	0.635	0.384	0.475	0.645

Data are expressed as means with SEM.

**Table 6:** Effect of different starch sources on plasma parameters of largemouth bass under high-temperature environment.

Groups	TP (g/L)	ALB (g/L)	ALT (U/L)	AST (U/L)
Corn starch	43.06 ± 1.06 ^ab^	15.96 ± 0.64	1.78 ± 0.25 ^b^	15.46 ± 1.28
Tapioca starch	40.11 ± 1.21 ^a^	14.83 ± 0.49	1.64 ± 0.17 ^b^	17.48 ± 2.67
Sweet potato starch	43.74 ± 1.34 ^b^	15.78 ± 0.36	1.35 ± 0.19 ^ab^	14.58 ± 2.48
Potato starch	45.05 ± 1.01 ^b^	14.94 ± 0.42	0.80 ± 0.15 ^a^	13.35 ± 0.98
Wheat starch	44.48 ± 0.85 ^b^	14.93 ± 0.79	1.03 ± 0.23 ^a^	15.92 ± 1.37
*p*-value	0.049	0.456	0.005	0.455

TP: total protein, ALB: albumin, ALT: alanine transaminase, AST: aspartate aminotransferase. Data are expressed as means with SEM. Values with different superscripts are significantly different (*p* < 0.05).

**Table 7:** Effect of different starch sources on antioxidant indices in the liver of largemouth bass under high-temperature environment.

Groups	CAT (U/mg Prot)	SOD (U/mg Prot)	MDA (nmol/mL)	GSH (μmol/g Prot)
Corn starch	0.95 ± 9.12 ^a^	0.31 ± 0.03 ^a^	0.20 ± 0.03 ^a^	1.56 ± 0.22 ^ab^
Tapioca starch	1.15 ± 0.19 ^ab^	0.30 ± 0.05 ^a^	0.37 ± 0.07 ^ab^	1.59 ± 0.28 ^ab^
Sweet potato starch	1.18 ± 0.06 ^ab^	0.35 ± 0.03 ^a^	0.50 ± 0.11 ^b^	0.91 ± 0.12 ^a^
Potato starch	1.32 ± 0.13 ^ab^	0.36 ± 0.02 ^ab^	0.35 ± 0.04 ^ab^	1.46 ± 0.50 ^ab^
Wheat starch	1.41 ± 0.11 ^b^	0.44 ± 0.01 ^b^	0.25 ± 0.06 ^a^	2.16 ± 0.27 ^b^
*p*-value	0.008	0.009	0.035	0.038

CAT: catalase, SOD: superoxide dismutase, MDA: malondialdehyde, GSH: glutathione. Data are expressed as means with SEM. Values with different superscripts are significantly different (*p* < 0.05).

## Data Availability

Data are contained within the article.
